# Gossypol Inhibits Non-small Cell Lung Cancer Cells Proliferation by Targeting EGFR^L858R/T790M^

**DOI:** 10.3389/fphar.2018.00728

**Published:** 2018-07-09

**Authors:** Yuwei Wang, Huanling Lai, Xingxing Fan, Lianxiang Luo, Fugang Duan, Zebo Jiang, Qianqian Wang, Elaine Lai Han Leung, Liang Liu, Xiaojun Yao

**Affiliations:** ^1^State Key Laboratory of Quality Research in Chinese Medicine, Macau University of Science and Technology, Macau, China; ^2^Department of Thoracic Surgery, Guangzhou Institute of Respiratory Health and State Key Laboratory of Respiratory Disease, The First Affiliated Hospital of Guangzhou Medical University, Guangzhou, China; ^3^Respiratory Medicine Department, Taihe Hospital, Hubei University of Medicine, Hubei, China

**Keywords:** gossypol, molecular docking, NSCLC, EGFR, TKI

## Abstract

**Background:** Overexpression of epidermal growth factor receptor (EGFR) has been reported to be implicated in the pathogenesis of non-small cell lung cancer (NSCLC). Several EGFR inhibitors have been used in clinical treatment of NSCLC, but the emergence of EGFR^L858R/T790M^ resistant mutation has reduced the efficacy of the clinical used EGFR inhibitors. There is an urgent need to develop novel EGFR^L858R/T790M^ inhibitors for better NSCLC treatment.

**Methods:** By screening a natural product library, we have identified gossypol as a novel potent inhibitor targeting EGFR^L858R/T790M^. The activity of gossypol on NSCLC cells was evaluated by cell proliferation, cell apoptosis and cell migration assays. Kinase activity inhibition assay and molecular docking were used to study the inhibition mechanism of gossypol to EGFR^L858R/T790M^. Western blotting was performed to study the molecular mechanism of gossypol inhibiting the downstream pathways of EGFR.

**Results:** Gossypol inhibited the cell proliferation and cell migration of NSCLC cells, and induced caspase-dependent cell apoptosis of NSCLC cells by upregulating the expression of pro-apoptotic protein BAD. Molecular docking revealed that gossypol could bind to the kinase domain of EGFR^L858R/T790M^ with good binding affinity through hydrogen bonds and hydrophobic interactions. Gossypol inhibited the kinase activity of EGFR^L858R/T790M^ with EC_50_ of 150.1 nM. Western blotting analysis demonstrated that gossypol inhibited the phosphorylation of EGFR and its downstream signal pathways in a dose-dependent manner.

**Conclusion:** Gossypol inhibited cell proliferation and induced apoptosis of NSCLC cells by targeting EGFR^L858R/T790M^. Our findings provided a basis for developing novel EGFR^L858R/T790M^ inhibitors for treatment of NSCLC.

## Introduction

Non-small cell lung cancer (NSCLC) accounts for approximately 85-90% of lung cancers, which has proven to be difficult to be treated due to poorly understood the pathogenesis (Oyewumi et al., 2014; Siegel et al., 2017). Conventional treatment strategies are used for NSCLC including surgical operation, radiotherapy and chemotherapy (Scott et al., 2007; Onishi et al., 2011; Uzel and Abacıoğlu, 2015). In addition, tyrosine kinase-based inhibitors (TKIs) molecular-targeted therapy are also employed to the treatment of NSCLC patients with EGFR mutations. Overexpression of EGFR has been reported and implicated in the pathogenesis of NSCLC, which account for more than 60% of NSCLC (Ohsaki et al., 2000). Therefore, it is increasing in clinic application as molecular targets for NSCLC patients with EGFR mutation.

The role of aberrant activation of the EGFR in NSCLC is well-documented (Sordella et al., 2004; Tracy et al., 2004; Gazdar and Minna, 2005; Sharma and Settleman, 2007; Sharma et al., 2007). The most common activating mutations, including point mutation L858R in exon 21 and deletions within exon 19 (del746-750) (Riely et al., 2006; Sharma et al., 2007), promote EGFR-driven cell proliferation and survival. Both first and second generation EGFR-targeted TKIs (gefitinib and erlotinib) targeting those activating mutants have been demonstrated to have a remarkable clinical response in the treatment of EGFR-mutated NSCLC (Lynch et al., 2004; Paez et al., 2004; Jackman et al., 2009; Rosell et al., 2009; Sequist et al., 2010). Although the early clinical results of first-generation EGFR inhibitors are impressive, unfortunately, most NSCLC patients with activating mutations eventually develop acquired resistance to EGFR inhibitors within several months. The most common mechanism of acquired resistance is the secondary T790M (gatekeeper residue Thr790 to methionine within the EGFR kinase domain) point mutation in exon 20 that occurs with an EGFR mutation (e.g., L858R), which accounts for approximately 60% in these acquired resistances (Balak et al., 2006; Kosaka et al., 2006; Yu et al., 2013). To overcome the acquired resistance to first-generation TKIs, several second- and third-generation EGFR TKIs [such as EKB-569 (Kwak et al., 2005), BIBW2992 (Li et al., 2008) and PF00299804 (Engelman et al., 2007)] have been developed. However, these agents still display limited clinical benefit for NSCLC patients with T790M mutation owing to dose-limiting toxicities (Oxnard et al., 2011; Miller et al., 2012). Recently, third-generation covalent EGFR inhibitor osimertinib (Ward et al., 2013; Cross et al., 2014) has been developed as mutant-selective EGFR inhibitor that specifically targeting EGFR^L858R/T790M^ mutation. However, the effective treatment of patients that harbor the EGFR T790M drug resistance mutation with osimertinib is limited by the emergence of new drug resistances to the tyrosine kinase inhibitor therapy (Thress et al., 2015; Büttner et al., 2017). C797S mutation was reported to be a major mechanism for resistance to third generation EGFR TKIs (Yu et al., 2015). In addition to C797S mutation, other rare tertiary EGFR mutations have also been reported, including novel solvent front mutations (G796S/R), hinge pocket mutations of the leucine residue at position 792 (L792F/H), binding interference at position 798 (L798I), and steric hindrance at position 718 (L718Q) (Bersanelli et al., 2016; Chabon et al., 2016; Chen et al., 2017; Ou Q. et al., 2017; Ou S.-H.I. et al., 2017). With the emergence of resistance mechanisms, there is an urgent need to discover a novel class of EGFR inhibitors that effectively inhibits drug-resistant EGFR^L858R/T790M^ mutation.

Natural products have been widely regarded as a pivotal source of leading compounds for drug development, recently, several natural products have been identified targeting EGFR^L858R/T790M^ to overcome resistance. (Jung et al., 2015; Xiao et al., 2016). In our previous studies, we have successfully identified several small molecules from natural products library that could inhibit the growth of gefitinib resistant NSCLC via different mechanisms. (Fan et al., 2015; Li et al., 2017). These compounds demonstrated significant anti-proliferative effects on a variety of NSCLC cell lines, including those with T790M and L858R/T790M mutations. In this study, we identified a small molecule gossypol from cottonseed, as a potent inhibitor targeting EGFR^L858R/T790M^. Gossypol and its derivatives exert antitumor effects on different cancer types *in vitro* and *in vivo*, including breast cancer (Xiong et al., 2017), colon cancer (Lan et al., 2015), chronic myeloid leukemia (Goff et al., 2013) and prostate cancer (Volate et al., 2010) by targeting MDM2, VEGFR, Bcl-2 and p53. Herein, the results from our work proved that gossypol could inhibit the proliferation of NSCLC cells by targeting EGFR^L858R/T790M^. Gossypol also inhibited the phosphorylation of EGFR and suppressed the phosphorylation of extracellular signal–regulated protein kinase (ERK) and AKT. These results indicated that gossypol could be developed as a new potent EGFR^L858R/T790M^ inhibitor and could inhibit the proliferation of NSCLC.

## Results and Discussion

### Gossypol Inhibits Cell Proliferation in NSCLC Cells

**FIGURE 1:**
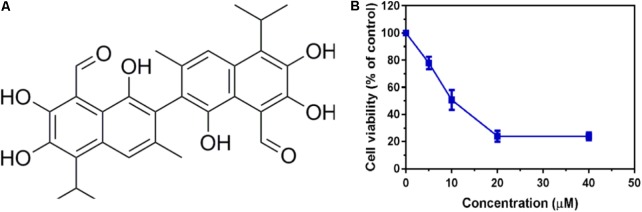
Cytotoxicity effect of gossypol on EGFR mutant cell line. **(A)** The structure of gossypol. **(B)** Evaluation of cell proliferation by gossypol in H1975 cells.

**FIGURE 2:**
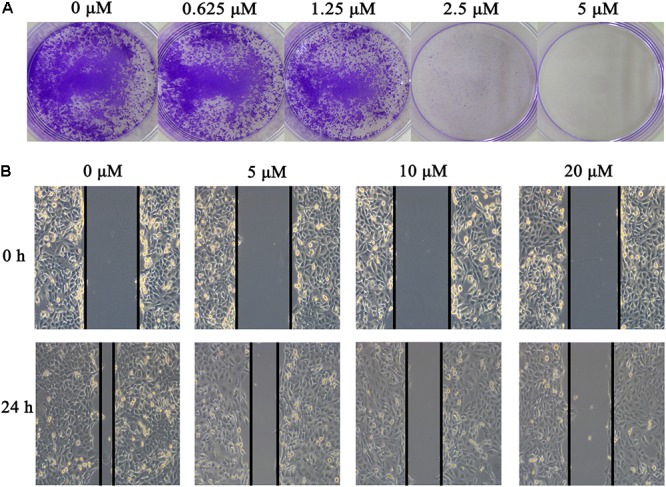
Effect of gossypol on H1975 cell line. **(A)** Colony formation of H1975 cells was monitored after gossypol (0–5 μM) treatment for 14 days, and photomicrographs of crystal violet stained colonies were depicted. **(B)** H1975 cells were treated with 0, 5, 10, and 20 μM for 24 h, and were analyzed for wound healing.

To identify potent small molecule inhibitor of EGFR^L858R/T790M^, we screened a natural products library with 235 compounds. We evaluated the anti-proliferative effect of each compound on H1975 cell line harboring EGFR^L858R/T790M^. Gossypol was identified and chosen for further mechanistic investigation due to its significantly anti-proliferative ability. H1975 cells were treated with an increasing concentration of gossypol for 72 h, and then cell viability was determined based on standard MTT assay protocol. As shown in **Figure 1**, the growth of H1975 cells were obviously inhibited by the treatment of gossypol in a dose-dependent manner, with 50% inhibition concentration (IC_50_) of 10.89 ± 0.84 μM. In addition, we have tested the cytotoxicity effect of gossypol on human normal lung fibroblast cell line CCD19 (IC_50_ is 14.89 ± 1.12 μM) and human NSCLC cell line H358 with EGFR^WT^ (IC_50_ is 35.26 ± 1.09 μM) (the corresponding results can be seen in Supplementary Figure S1). Afatinib was used as positive control (IC_50_ = 170.4 ± 1.1 nM). The structure and corresponding cytotoxicity of gossypol were showed in **Figure 1**. We also examined the effect of gossypol on cell colony formation (**Figure 2A**), in accordance with the cell cytotoxicity, gossypol significantly inhibited the colony formation capacity in a dose-dependent manner in H1975 cell line. Collectively, these results suggested that gossypol could inhibit the proliferation of H1975 cell line.

### Gossypol Induces Cell Apoptosis in NSCLC Cells

**FIGURE 3:**
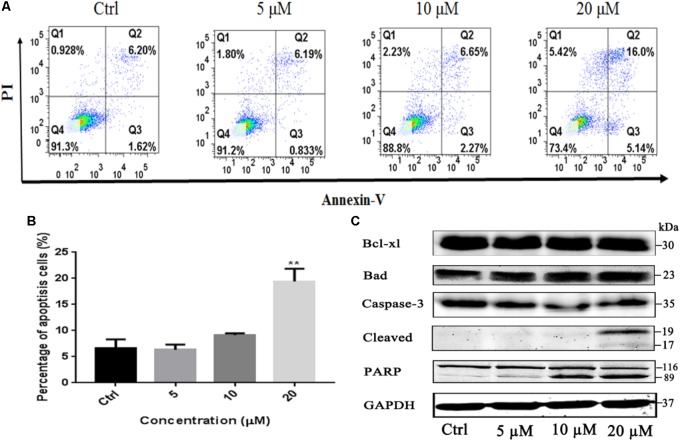
Apoptosis effect of Gossypol on H1975 cells. Flow cytometric analysis of cell apoptosis with gossypol at different concentrations (0, 5, 10, and 20 μM) for 24 h was determined. **(A)** Flow cytometry analysis of the apoptosis levels of h1975 cells after treatment with gossypol for 24 h. **(B)** Data from **(A)** were statistically analyzed. Mean ± SE. ^∗∗^*P* < 0.01. **(C)** Western blot analysis of apoptotic markers of H1975 cells after treatment of gossypol for 24 h.

To investigate whether the induction of apoptosis also contributed to gossypol-mediated growth inhibition of H1975 cells, Annexin V-FITC/PI staining assay was employed to analyze the number of apoptotic cells after treatment with gossypol using a flow cytometer. As shown in **Figures 3A,B**, gossypol induced cell apoptosis on H1975 cell line with a concentration-dependent manner.

Bcl-2 family members play key roles in the regulation of apoptotic progress. To understand how gossypol induced apoptosis, we next examined whether gossypol could alter the expression of apoptotic proteins in H1975 cells. As shown in **Figure 3C** and Supplementary Figure S4, treatment with gossypol for 24 h remarkably upregulated the expression level of proapoptotic protein Bad in a concentration-dependent manner. Moreover, we also observed that gossypol induced PARP cleavage, a hallmark of caspase-dependent apoptosis, in accordance with the expression level of cleaved caspase-3. Therefore, these results suggested that gossypol induced caspase-dependent apoptotic cell death by upregulating the expression of pro-apoptotic protein Bad in NSCLC cells.

### Gossypol Inhibits the Cell Migration of H1975 Cell Line

The effect of gossypol on H1975 cell migration capability was estimated by a wound-healing assay. In the wound-healing assay (see **Figure 2B**), cells treated with gossypol reduced the rate of wound healing along with the increasing of treatment concentration, which was significantly lower than the untreated cells following incubation. These results demonstrated that gossypol inhibited the migration ability of H1975 cell lines in a dose-dependent manner.

### Gossypol Inhibits the Activity of Tyrosine Kinase

To assess the kinase inhibition activities of gossypol, we performed a kinase inhibition profile assay of gossypol against recombinant human EGFR^L858R/T790M^. The selected compound gossypol exhibited inhibitory activity, which effectively inhibited the enzymatic activity of EGFR^L858R/T790M^ with an EC_50_ value of 150 ± 30.7 nM (see Supplementary Figure S2). Besides, gossypol also inhibited the enzymatic activity of EGFR^WT^ with an EC_50_ value of 252.9 ± 26.9 nM, higher than that to EGFR^L858R/T790M^ (the corresponding results can be seen in Supplementary Figure S2). Afatinib was used as positive control (EC_50_ = 9.6 ± 2.9 nM). The effect of gossypol on cells is very complicated, and it is still difficult to distinguish which part is caused by EGFR targeting. To ensure the consistency of the experimental results, we conducted the entire ELISA enzyme inhibiting assay at the same time. Therefore, EGFR^WT^ could be used as control to compare with EGFR^L858R/T790M^.

### Molecular Docking Predicts the Potential Binding of Gossypol to EGFR

**FIGURE 4:**
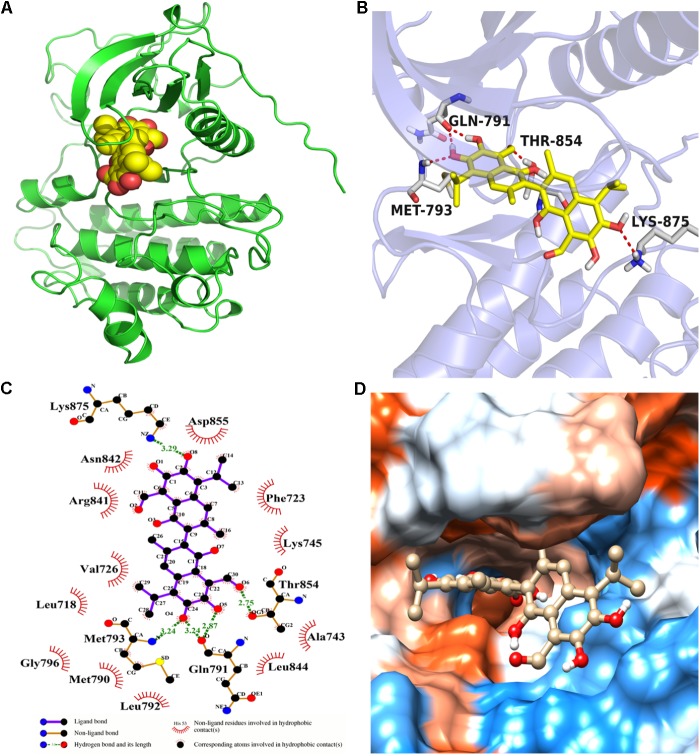
The binding mode between gossypol and EGFR^L858R/T790M^ protein. **(A)** The 3D structure of EGFR^L858R/T790M^. **(B)** Gossypol was docked into the EGFR kinase domain, showing interactions between gossypol and key residues. **(C)** A two-dimensional interaction map of gossypol and EGFR. **(D)** The hydrophobic surface of EGFR^L858R/T790M^.

Molecular docking calculation was performed to gain insight into the binding mode between gossypol and EGFR^L858R/T790M^. The molecular docking results (see **Figure 4** and Supplementary Figure S3) proved that gossypol could be docked into the kinase domain mainly composed of hydrophobic residues of C-helix and A-loop with a docking score of -6.42 ± 0.24 kcal/mol. Five hydrogen bonds were formed between gossypol and the carbonyl group of Q791, amino group of M793, hydroxyl group of T854 and amino group of K875. In addition, the hydrophobic contacts formed between gossypol and surrounded residues, including L718, M790, F723, F858, L792, L844, and M793, which also contributed to the interaction between gossypol and EGFR^L858R/T790M^. Therefore, the above results suggested that gossypol could bind to EGFR^L858R/T790M^.

### Gossypol Effectively Suppresses Phosphorylation of EGFR as Well as Its Downstream Signaling Pathway

**FIGURE 5:**
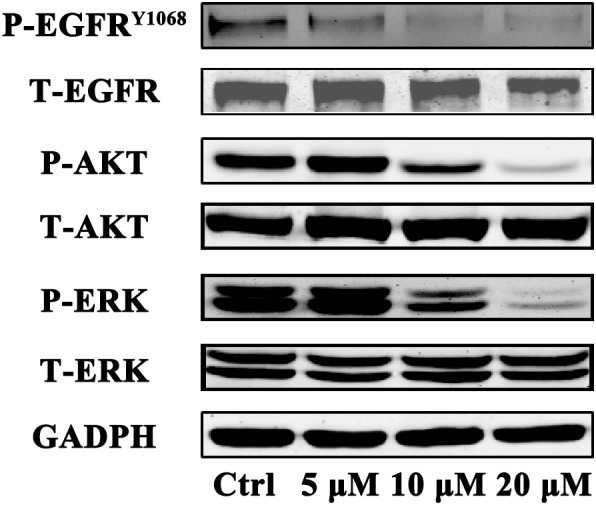
Immunoblot analysis of p-EGFR, EGFR, p-AKT, AKT, p-ERK, and ERK in H1975 cell after treatment with gossypol for 24 h. GAPDH was used as a loading control.

To determine whether gossypol could inhibit the expression level of EGFR in cells, we investigated the effect of gossypol on the phosphorylation of EGFR in NSCLC cells. H1975 cells were treated with gossypol (0–20 μM) for 24 h. Western blot analysis showed that gossypol inhibited the phosphorylation of EGFR (Tyr 1068) in a concentration dependent manner (see **Figure 5**). To explore the detailed anti-cancer mechanism of gossypol, we further evaluated the downstream pathways of EGFR, including ERK and AKT signaling pathways. Treatment with gossypol also inhibited the phosphorylation of AKT and ERK in a concentration-dependent manner, consistent with the tendency of phosphorylation level of EGFR. Thus, our results indicated that gossypol could suppress the phosphorylation of EGFR and its downstream AKT and ERK signaling pathways, resulting in induction of apoptosis and proliferation inhibition of H1975 cells.

## Conclusion

In this study, by screening a natural products library, we have identified that gossypol was a potential anticancer agent targeting EGFR^L858R/T790M^. Our results proved that gossypol inhibited the proliferation and induced apoptosis of human NSCLC cell line harboring EGFR^L858R/T790M^. Moreover, gossypol decreased the phosphorylation level of EGFR and its downstream signaling pathways AKT and ERK. Overall, our findings indicate that gossypol is a novel potent EGFR^L858R/T790M^ inhibitor, which may serve as a useful therapeutic agent against NSCLC harboring EGFR^L858R/T790M^ mutation.

## Materials and Methods

### Reagents

Gossypol was purchased from Selleck Ltd., which was dissolved in dimethyl sulfoxide (DMSO) to form a 20 mM stock solution. Fetal bovine serum (FBS), antibiotics and RPMI medium were purchased from Gibco (Carlsbad, CA, United States). RIPA lysis buffer and antibodies Bad, Bcl-XL, PARP, Cleaved Caspase-3, anti-p-EGFR (1068), anti-p-extracellular signal-regulated kinase 1/2 (Erk1/2) (Thr202/Tyr204), anti-p-Akt (Ser473), anti-Erk1/2, anti-Akt, anti-PERK, and anti-EGFR were purchased from Cell Signaling Technology (Beverly, MA, United States). Anti-GAPDH was purchased from Santa Cruz (Dallas, TX, United States).

### Cell Culture

The human NSCLC cell line H1975 was purchased from the American Type Culture Collection (ATCC) (Manassas, VA, United States). Cells were cultured in RPMI1640 medium supplemented with 10% FBS, 100 U/ml penicillin and 100 μg/ml streptomycin. All the cells were cultured at 37°C in a humidified atmosphere containing 5% CO_2_.

### Cell Proliferation Assay

Cell viability was evaluated by using the standard 3-(4,5-dimethylthiazol-2-yl)-2,5-diphenyltetrazolium bromide MTT assay. Briefly, 3 × 10^3^ cells per well were plated in 96-well plates and cultured overnight for cell adhesion. The cells were treated with DMSO or various concentrations of gossypol for 72 h. Subsequently, 10 μL MTT was added into each well and incubated for 4 h, and then the dark blue crystals were dissolved with 100 μl of the resolved solution (99% DMSO). Finally, the absorbance at 570 nm was measured by microplate reader (Tecan, Morrisville, NC, United States). The cell viability was calculated relative to controls, with results based on at least three independent experiments. Cells treated with the vehicle (DMSO) alone served as a control.

### Colony Formation Assay

Briefly, H1975 cells were seeded in 6-well plates (1000 cells/well), after attachment overnight, cells were exposed to various concentration of gossypol with medium changes every 3 days until visible colonies formed. The colonies were washed with cold PBS, then fixed in 4% paraformaldehyde (PFA) for 15 min, and then stained with 0.5% crystal violet (1% PFA, 0.5% crystal violet, and 20% methanol in ddH_2_O) for 20 min. The colonies were photographed.

### Apoptosis Analysis Assay

NSCLC cells were plated on 6-well plate with cell density of 2 × 10^5^ cells per well and cultured overnight for adhesion. Subsequently, the cells were treated with different concentrations of gossypol for 24 h. After treatment, the cells were harvested by trypsin digestion and washed twice with ice-cold PBS, and resuspended in 100 μl 1 × binding buffer. Next, 4 μl of propidium iodide (PI, 1 mg/ml) and 1 μl Annexin-V fluorescein dye were added to the solution and mixed well at room temperature in the dark for 15 min. After that, the cells were resuspended in 300 μl of 1 × binding buffer from BD Biosciences (San Jose, CA, United States). The percentage of apoptotic cells was quantitatively measured using a BD FACSAria III flow cytometer from BD Bioscience (San Jose, CA, United States).

### Enzyme-Linked Immunosorbent Assay (ELISA)

The kinase activity was evaluated with ELISA assay based on the kinase domain of dual-mutant EGFR (EGFR^L858R/T790M^) recombinant human protein (Peng et al., 2014). Briefly, 20 μg/mL Poly (Glu, Tyr) 4:1 (Sigma, St. Louis, MO, United States) was precoated in 96-well plates as substrate. Active kinases were added and incubated with indicated gossypol in 1 × reaction buffer containing 5 μmol/L ATP at 37°C for 1 h. Then, the wells were washed with PBS and then incubated with an anti-phosphotyrosine (PY99) antibody (Santa Cruz Biotechnology, Santa Cruz, CA, United States) followed by a horseradish peroxidase (HRP)-conjugated secondary antibody. The wells were read with a multiwell spectrophotometer (VERSAmax^TM^, Molecular Devices, Sunnyvale, CA, United States) at 492 nm. The inhibitory rate (%) was calculated with the following formula: [1–(A_492_ treated/A_492_ control)] × 100%, and responding EC_50_ values were calculated from the fitting inhibitory curves.

### Molecular Docking

The X-ray structure of EGFR^L858R/T790M^ with a resolution of 2.5 Å complexed with diaminopyrimidine derivative was retrieved from the Protein Data Bank [PDB ID code 4RJ8 (Hanan et al., 2014)] for docking with gossypol. Molecular structures were prepared using the standard procedure from the Protein Preparation Wizard module in Schrödinger 2015. The docking grid box was defined using the Receptor Grid Generation tool in Glide by centering on native ligand in the EGFR^L858R/T790M^ structure. The structure of gossypol was derived from the PubChem database^1^, which was imported to the LigPrep module (Version 2.3, Schrödinger, LLC, New York, NY, United States) based on OPLS-2005 force field (Kaminski et al., 2001). The ionized state was assigned by using Epik (Version 2.0, Schrödinger, LLC, New York, NY, United States) at a pH value of 7.0 ± 2.0. Gossypol was docked into the kinase domain of the EGFR^L858R/T790M^ using the Glide (Version 5.5, Schrödinger, LLC, New York, NY, United States) with the extra precision (XP) scoring mode. In the process of molecular docking, 5000 poses were generated during the initial phase of the docking calculation. The best binding pose for Gossypol was conserved for the further analysis.

### Western Blot Analysis

Preparation of whole-cell protein lysates for western blot analysis was conducted as follows. After treatment, cells were lysed in RIPA lysis buffer (150 mmol/L NaCl, 50 mmol/L Tris–HCl, pH 8.0,1% Triton X-100, 0.1% SDS, and 1% deoxycholate) containing protease inhibitor cocktail from Roche (Basel, Lewes, United Kingdom) for 15 min on ice and then boiled for 10 min. The concentration of total protein was determined with a Bio-Rad DCTM Protein Assay Kit (Bio-Rad, Hercules, CA, United States). Equal amounts of total protein (30 μg) protein lysate were loaded and separated by 10% SDS–polyacrylamide gel electrophoresis and then transferred to a nitrocellulose (NC) membrane from Millipore (Billerica, MA, United States). The membranes were blocked with 5% milk without fat in 1 × TBST for 2 h at room temperature, and then incubated with various primary antibodies, including phospho-AKT, phospho-ERK, t-AKT, t-ERK, phospho-EGFR (Tyr1068), t-EGFR at 1:1000 dilutions and anti-GADPH antibody at a 1:800 dilution overnight at 4°C. After washing the membranes in TBST three times (5 min per time), secondary fluorescent antibodies, either anti-rabbit or anti-mouse secondary antibodies depending on the source of the primary anti-bodies, were added to the membrane at 1:10,000 dilutions at room temperature for 2 h. GAPDH was used as the loading control and for normalization. The signal intensity of the membranes was detected using an LI-COR Odessy scanner (Belfast, ME, United States).

### Statistical Analysis

The results were expressed as mean values ± standard error (mean ± SE). Statistical analysis was performed using one-way ANOVA followed by Bonferroni’s post-tests. Significance was accepted at *P* < 0.05.

## Author Contributions

EL, LL, and XY conceived this research, led the project, and revised the manuscript. YW, HL, XF, FD, ZJ, QW, and LL carried out the experiments and analyzed the data. YW and XY wrote the manuscript. All authors reviewed the manuscript.

## Conflict of Interest Statement

The authors declare that the research was conducted in the absence of any commercial or financial relationships that could be construed as a potential conflict of interest.

## Footnotes

1 http://pubchem.ncbi.nlm.nih.gov
