# Supplementary material for: Gossypol Inhibits Non-small Cell Lung Cancer Cells Proliferation by Targeting EGFRL858R/T790M
Source: Front Pharmacol. 2018 Jul 9;9:728. doi: 10.3389/fphar.2018.00728 (PMC6046546; doi:10.3389/fphar.2018.00728)
Supplement: Supplementary file 1 [file Presentation_1.pdf]

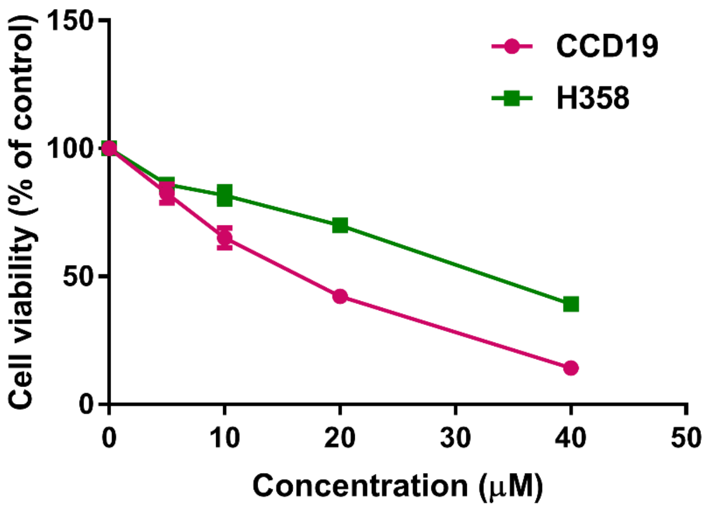

Supplementary Figure S1. Cytotoxicity effect of gossypol on human normal lung fibroblast cell line CCD19 and human NSCLC cell line H358 with EGFR WT. Cytotoxicity effect of gossypol on CCD19 and H358 cell lines after 72h treatment. Pink lines represents CCD19 cell line with IC_{50} of 14.89±1.12 μM, while green line represents H358 cell line with IC_{50} of 35.26±1.09 μM.

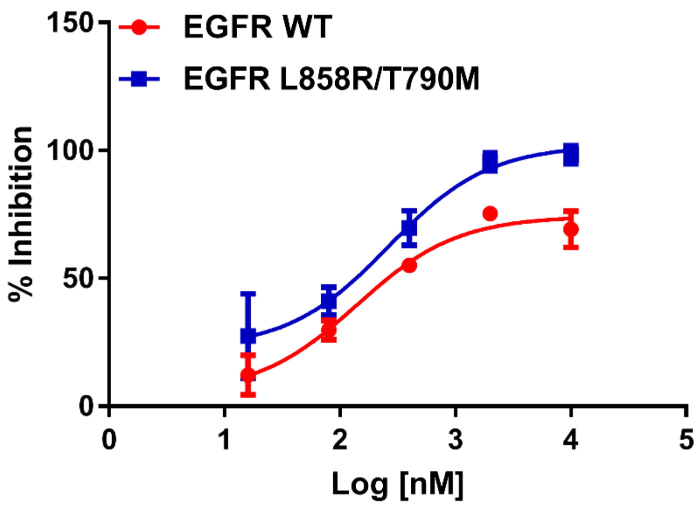

Supplementary Figure S2. Gossypol inhibited EGFR kinase activity. The kinase profiling assay was conducted as described in the Experimental section by using ELISA kinases assay. EC_{50} values were obtained from three independent experiments and expressed as mean ± SD. Red line represents EGFR WT with EC_{50} of 252.9±26.9 nM, while blue line represents $EGFR^{L858R/T790M}$ with EC_{50} of 150.1±30.7 nM.

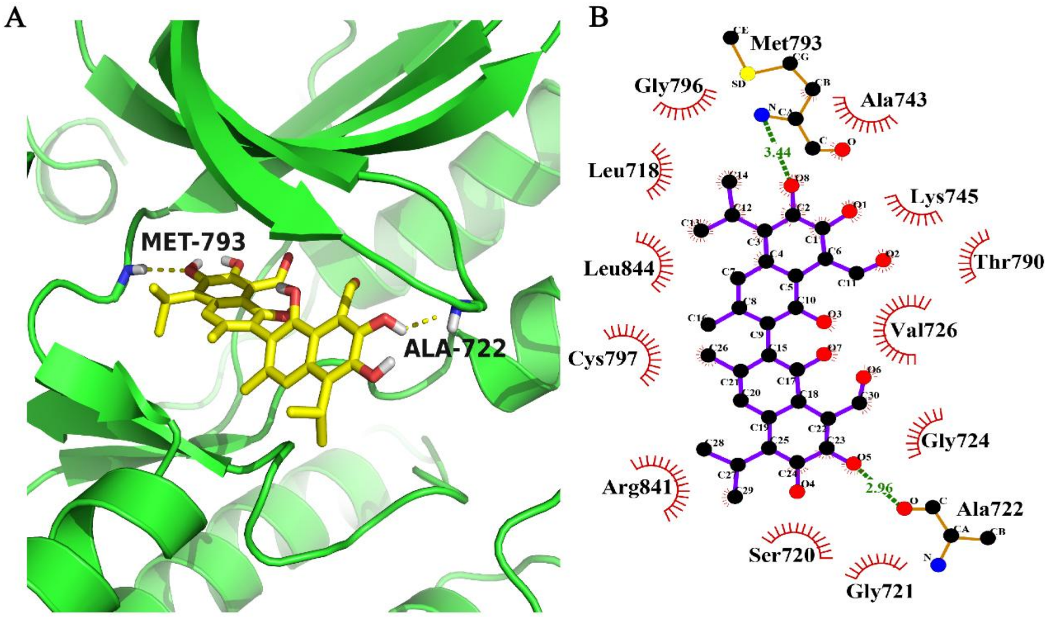

Supplementary Figure S3. The binding mode between gossypol and the kinase domain of EGFR^{WT} protein. (A): Gossypol was docked into the EGFR^{WT} kinase domain with docking score of -6.30 kcal/mol, showing interactions between gossypol and key residues. (B): A two-dimensional interaction map of gossypol and EGFR^{WT}.

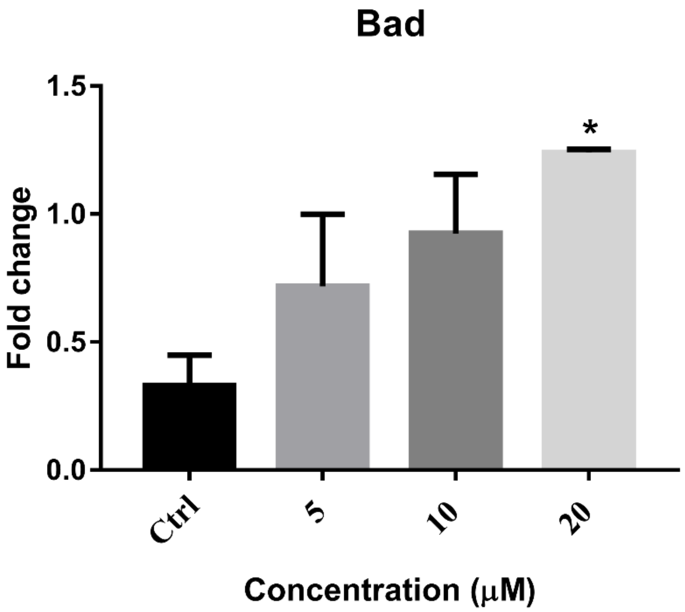

Supplementary Figure S4. Fold change of bad protein expression. Data is presented as the mean±SEM. Statistical analysis was performed with one-way ANOVA. ∗$p<0.05$.
